# Supplementary material for: In Vitro Metabolism of Helenalin Acetate and 11α,13-Dihydrohelenalin Acetate: Natural Sesquiterpene Lactones from Arnica
Source: Metabolites. 2022 Jan 17;12(1):88. doi: 10.3390/metabo12010088 (PMC8780470; doi:10.3390/metabo12010088)

Supplementary Materials

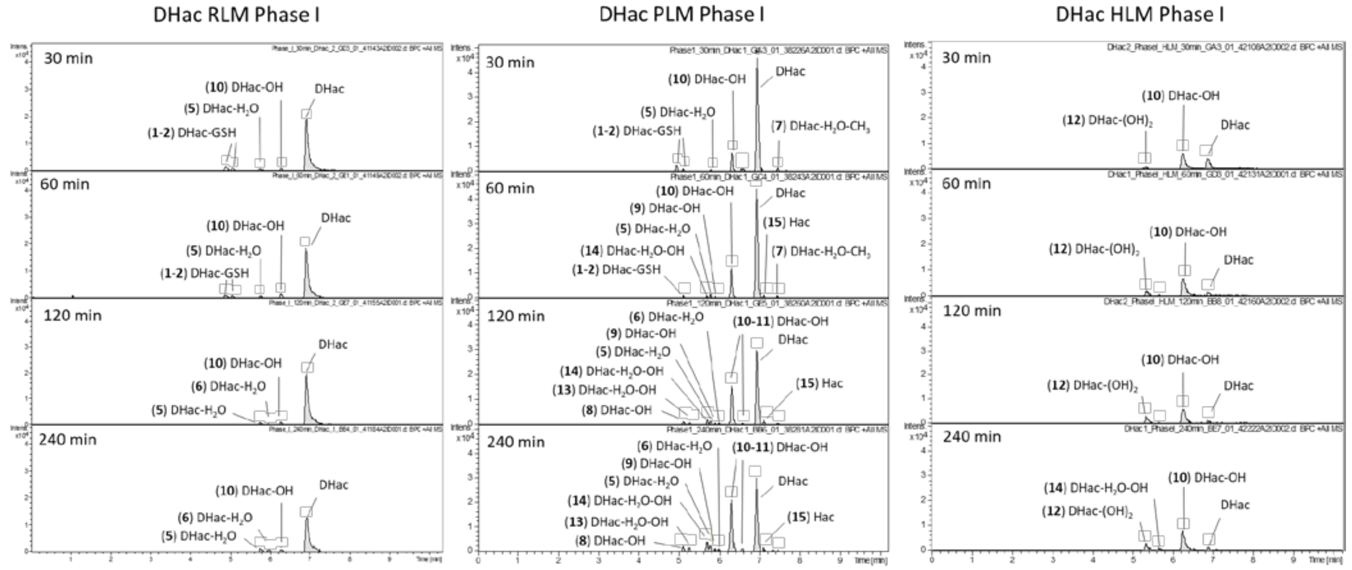

Figure S1: Difference chromatograms of DHac after incubation with RLM, PLM or HLM and NADPH regenerating system (only phase I) for 30 min, 60 min, 120 min and 240 min, respectively.

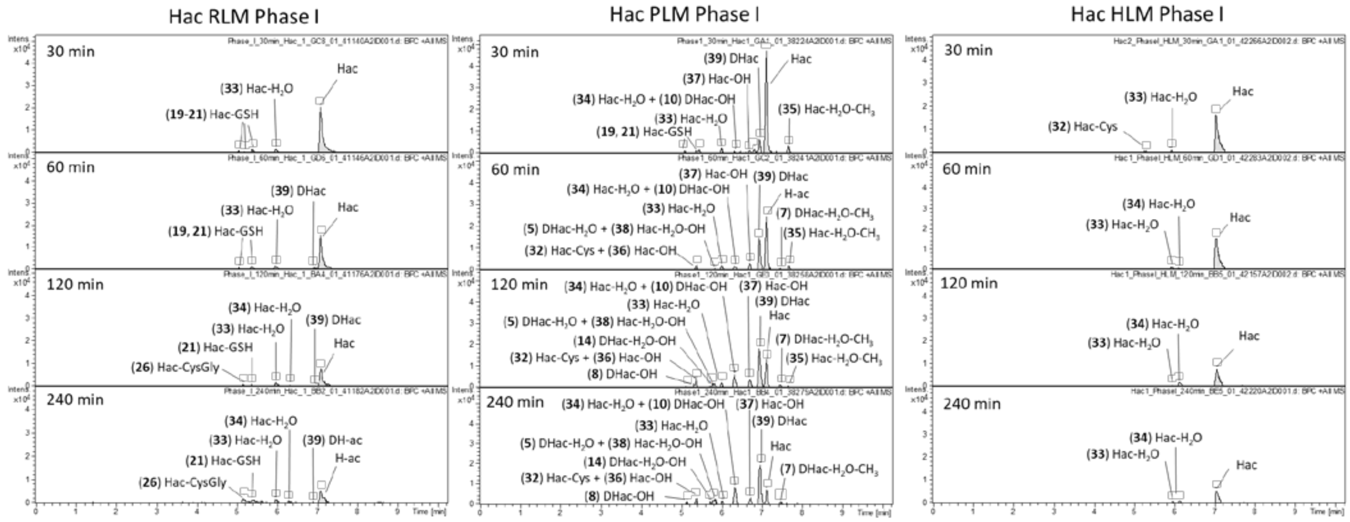

Figure S2: Difference chromatograms of Hac after incubation with RLM, PLM or HLM and NADPH regenerating system (only phase I) for 30 min, 60 min, 120 min and 240 min, respectively.

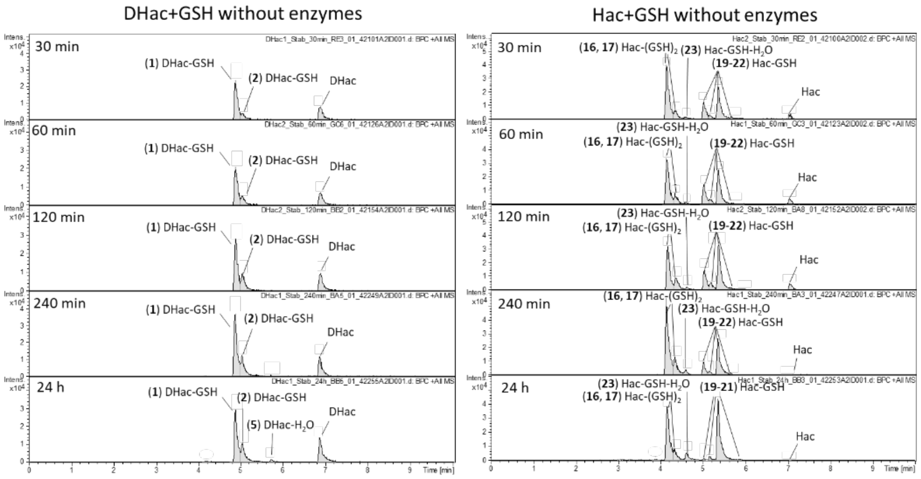

Figure S3: Difference chromatograms of DHac and Hac after incubation with GSH and NADPH regenerating system without GST and microsomes (stability control for spontaneous reactions) for 30 min, 60 min, 120 min, 240 min and 24 , respectively.

Identification of metabolites (**1**)-(**39**)

In this supplement, structure elucidation based on monoisotopic masses of pseudo-molecular ions and fragments, shifts in retention time and previously published data about the reactivity of DHac and Hac with GSH and Cys is described. For the LC-MS chromatograms, please compare Figures 2 and 4 in the main document. MS/MS spectra of the product ion scans with the corresponding metabolite as precursor are shown in the following subsections. In addition, possible fragmentation sites are marked and calculated *m/z* values are given next to the structures for comparison with the measured values. In order to easily follow the discussion about possible connection sites, the structures of DHac and Hac are shown with numbered carbons.

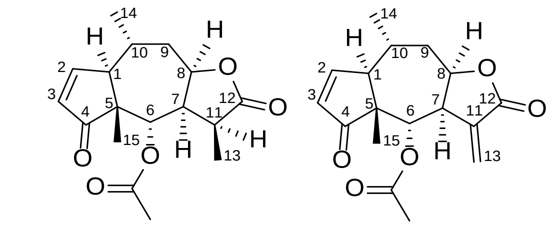

11α,13-Dihydrohelenalin acetate (DHac) — Helenalin acetate (Hac)

(**1**, **2**) DHac-GSH

For DHac two GSH-conjugates with $[M+H]^+$ at *m/z* 614.2378 were detected. Two fragments of the MS/MS spectra (*m/z* 539.2262 (**1**) and *m/z* 485.1996 (**2**)) result from neutral loss of glycine ($C_2H_5NO_2$, 75.0325 Da) or glutamic acid ($C_5H_9NO_4$, 129.0426 Da), respectively. Fragment *m/z* 425.1779 (**2**) is formed by neutral loss of acetic acid ($C_2H_4O_2$, 60.0265 Da) from the latter and *m/z* 247.1394 (**1**) and *m/z* 247.1347 (**2**) represent the DHac core fragment after cleavage of GSH ($C_{10}H_{17}N_3O_6S$, 307.0838 Da) and acetic acid. Other fragments of this metabolites result from the GSH moiety: *m/z* 308.0885 (**1**) and *m/z* 308.0939 (**2**) represent protonated free GSH whereas *m/z* 179.0488 (**1**) and *m/z* 179.0496 (**2**) were formed by neutral loss of glutamic acid from GSH. Based on MS/MS spectra, no differentiation between the two GSH-conjugates is possible because the observed fragmentation is possible for both metabolites each. Nevertheless, it is known from literature that GSH reacts with the Michael acceptor of the molecule leading to a conjugation at C-2. It was shown that the 2β-*S*-GS-

conjugate is favoured in spontaneous reactions [16]. Since (**1**) is formed in higher amounts compared with (**2**) it is most likely that (**1**) is the 2β-epimer (DHac-2βGSH) and (**2**) the 2α-epimer (DHac-2αGSH).

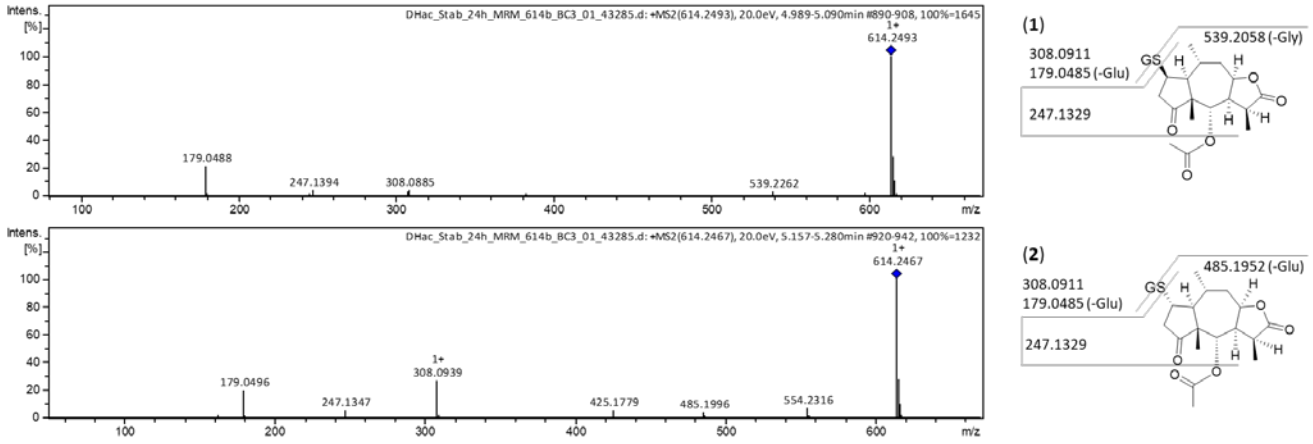

(**3**, **4**) DHac-GSH-OH

Two hydroxylated GSH-conjugates with $[M+H]^+$ at *m/z* 630.2327 were identified after incubation with HLM for 30 min. At this time, only GSH-conjugates (**1**) and (**2**) as well as one hydroxide (**10**) was formed. This leads to the assumption that (**3**) and (**4**) are GSH-conjugates as discussed for (**1**) and (**2**) and the position of the hydroxide is most likely at C-11 as shown for (**10**). In analogy to (**1**) and (**2**) the 2β-*S*-GSH-epimer (**3**, DHac-2βGSH-11OH) elutes earlier and was formed in higher amounts compared with the 2α-*S*-GSH-epimer (**4**, DHac-2αGSH-11OH). Regarding fragmentation, the fragments *m/z* 308.0788 (**3**), *m/z* 308.1068 (**4**), *m/z* 179.0444 (**3**) and *m/z* 179.0789 (**4**) of protonated GSH are developed as for (**1**) and (**2**). The fragment *m/z* 245.1341 (**3**) and *m/z* 245.0719 (**4**) result from cleavage of GSH and water.

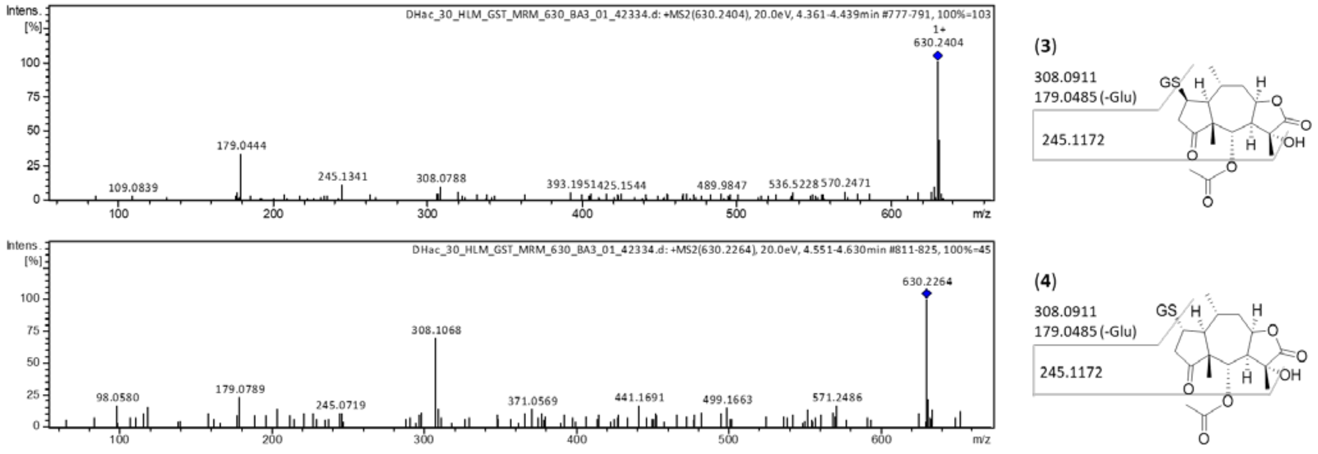

(**5**-**6**) DHac-H_2O

Two metabolites with $[M+H]^+$ at *m/z* 325.1646, $[M+Na]^+$ at *m/z* 347.1465 and $[M+K]^+$ at *m/z* 363.1204 were identified as results of the addition of water. The most probable position for water addition is the Michael acceptor (C-2 conjugation) as in many biological reactions [31]. Another possibility is hydration at the lactone moiety [32]. Both observed metabolites have similar retention times (Rt = 5.8 min and 6.0 min) and similar fragments at first glance: *m/z* 265.1445 (**5**) and *m/z* 265.1446 (**6**) result from loss of acetic acid, *m/z* 247.1366 (**5**) and *m/z* 247.1158 (**6**) from additional dehydration and the fragments *m/z* 229.1231 (**5**), *m/z* 229.1075 (**6**), *m/z* 201.1300 (**5**) and *m/z* 201.1198 (**6**) are typical DHac fragments as described in the main text. Nevertheless,

there are also some differences: *m/z* 279.1138 (neutral loss of formic acid CH_2O_2, 46.0055 Da) was only formed for (**6**), which makes sense if (**6**) is hydrolysed at the lactone moiety (DHac-12H_2O). Similarly, *m/z* 219.1399, the most intense fragment of (**5**), was not found in case of (**6**), because it would have to be formed by cleavage of CO (after loss of acetic acid and water, i.e. from *m/z* 247.1366) which is not feasible from a carboxylic acid group. Consequently, (**5**) is identified as DHac-2H_2O.

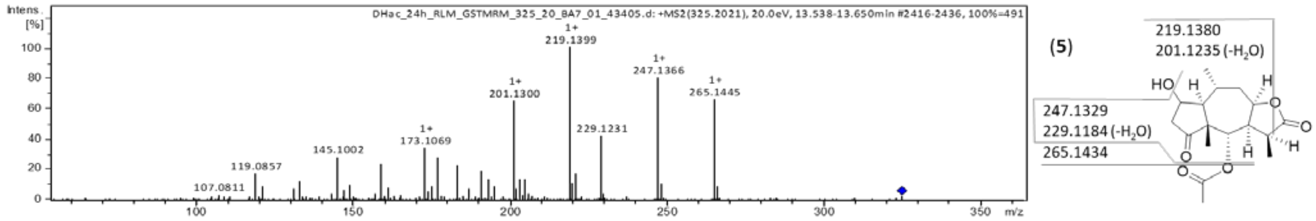

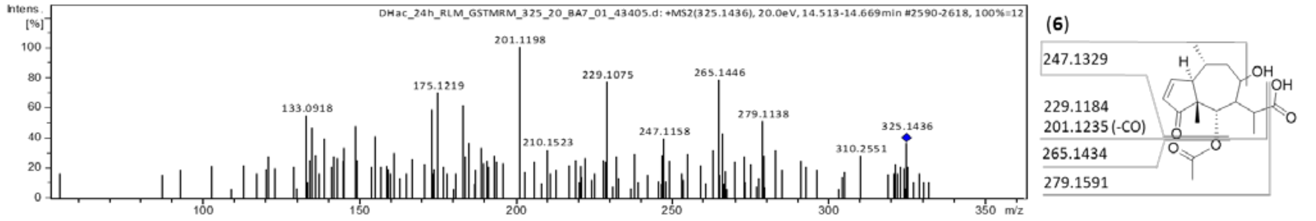

(**7**) DHac-H_2O-CH_3

Metabolite (**7**) was identified as formal methanol adduct (or, more likely in the context of the conducted metabolism experiments, methylated water adduct) based on accurate mass of the adduct ions ($[M+H]^+$ at *m/z* 339.1802, $[M+Na]^+$ at *m/z* 361.1622 and $[M+K]^+$ at *m/z* 377.1361). Since this is only a minor metabolite occurring in very low concentrations, MS/MS fragmentation did not result in enough ions to give information on the position of the methylation. For this metabolite the two water adducts (**5**) and (**6**) are possible precursors. In analogy with Hac, which has no metabolite with hydrolysed lactone moiety but also a methylated water adduct, methylation is most likely at the water conjugate at C-2, i.e. on metabolite (**5**), leading to DHac-2H_2O-CH_3.

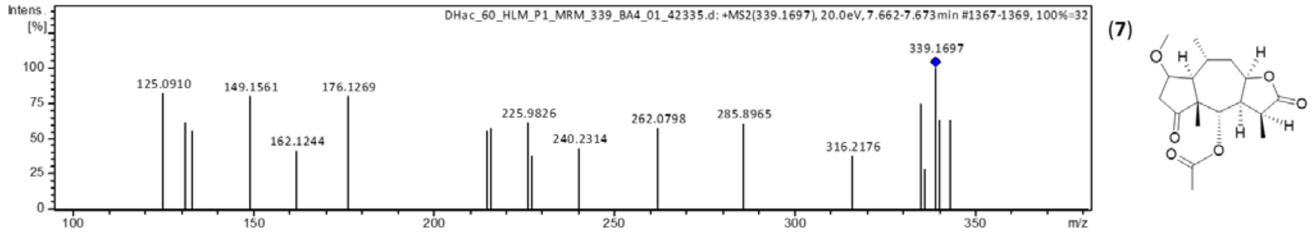

(**8**, **9**, **10**, **11**) DHac-OH

Four metabolites (**8**), (**9**), (**10**), (**11**) with $[M+H]^+$ at *m/z* 323.1489, $[M+Na]^+$ at *m/z* 345.1309 and $[M+K]^+$ at *m/z* 361.1048 were detected. The difference of 15.9949 Da compared with the parent compound DHac can be explained by addition of one oxygen atom, i.e. hydroxylation or epoxidation of a double bond. For DHac epoxidation is possible at C-2/ C-3 and would result in fragments at *m/z* 263.1121 by neutral loss of acetic acid, followed by deoxygenation resulting in *m/z* 247.1329. This combination could not be observed for metabolites (**8**-**11**). Another common biotransformation reaction is hydroxylation which is likely catalysed by CYP 450 [29]. A preference of CYP 450 for tertiary rather than secondary and primary carbons was reported [30]. Considering reactive sites of DHac, tertiary carbons in α-position to functional groups (C-1, C-6, C-8, C-11) are most likely hydroxylated because of inductive effects. Hydroxylation at C-6 can be excluded for (**8**-**11**) because fragment *m/z* 263.1121 followed by *m/z* 247.1329 would be expected for a hydroxide at C-6.

To make a tentative assignment which peak best represents which metabolite, the MS/MS spectra were analysed. Metabolite (**10**) forms the fragments *m/z* 263.1377 (resulting from neutral loss of acetic acid) and *m/z* 245.1248 (by subsequent dehydration). Further fragments result from loss of acetic acid and CO (*m/z* 235.1378) as well as dehydration of the latter once (*m/z* 217.1293) or twice (*m/z* 199.1152). This metabolite has the same retention time as the Hac water conjugate at C-13 (**34**, Rt = 6.3 min). For that reason, (**10**, Rt = 6.3 min) is being considered as the hydroxide at C-11 (DHac-11OH) which has a very similar structure compared with (**33**). MS/MS fragments support this hypothesis because the fragments at *m/z* 175.1119 ($[M+H-C_2H_4O_2-H_2O-CO-C_2H_2O]^+$) and *m/z* 147.1167 (by loss of CO from the latter) are only formed if the hydroxide is cleaved with C-11/C-13 as hydroxyacetylene (C_2H_2O).

Other activated tertiary carbons are C-1 (α-position to cyclopentenone system) and C-8 (α-position to lactone moiety). The fragment *m/z* 261.1134 of metabolite (**11**) might result from neutral loss of CO_2 and dehydrogenation, most likely at the lactone moiety if C-8 is hydroxylated. Consequently, (**11**) is most likely hydroxylated at C-8 (DHac-8OH). Metabolite (**8**) shares the fragments *m/z* 263.1385 (after neutral loss of acetic acid), *m/z* 245.1208 (dehydration of the latter), *m/z* 217.1296 (neutral loss of acetic acid, CO and water) and *m/z* 199.1154 (dehydration of *m/z* 217.1296) with metabolite (**10**). Under consideration of the four expected possible hydroxylation sites, (**8**) would then have to be hydroxylated at C-1 (DHac-1OH).

The MS/MS spectra of (**9**) shows fragments at *m/z* 265.1539 and *m/z* 247.1517 which indicate loss of $C_2H_2O_2$ (58.0055 Da) followed by dehydration. Usually, neutral loss of acetic acid ($C_2H_4O_2$, 60.0265 Da) is the first step in DHac fragmentation. This process is not likely to occur if C-7 is hydroxylated since it requires a proton at C-7 to be included in the acetic acid molecule. In case of (**9**), a loss of $C_2H_2O_2$ (58.0055 Da) instead of $C_2H_4O_2$ is observed. Hence, (**9**) could be assumed to be hydroxylated at C-7. For this structure (DHac-7OH), the fragments *m/z* 229.1344 ($[M+H-C_2H_4O_2-H_2O]^+$), 201.1304 ($[M+H-C_2H_4O_2-H_2O-CO]^+$), *m/z* 173.1030 ($[M+H-C_2H_4O_2-H_2O-CO-C_2H_4]^+$) and *m/z* 145.1008 ($[M+H-C_2H_4O_2-H_2O-2\ CO-C_2H_4]^+$) are in common with DHac.

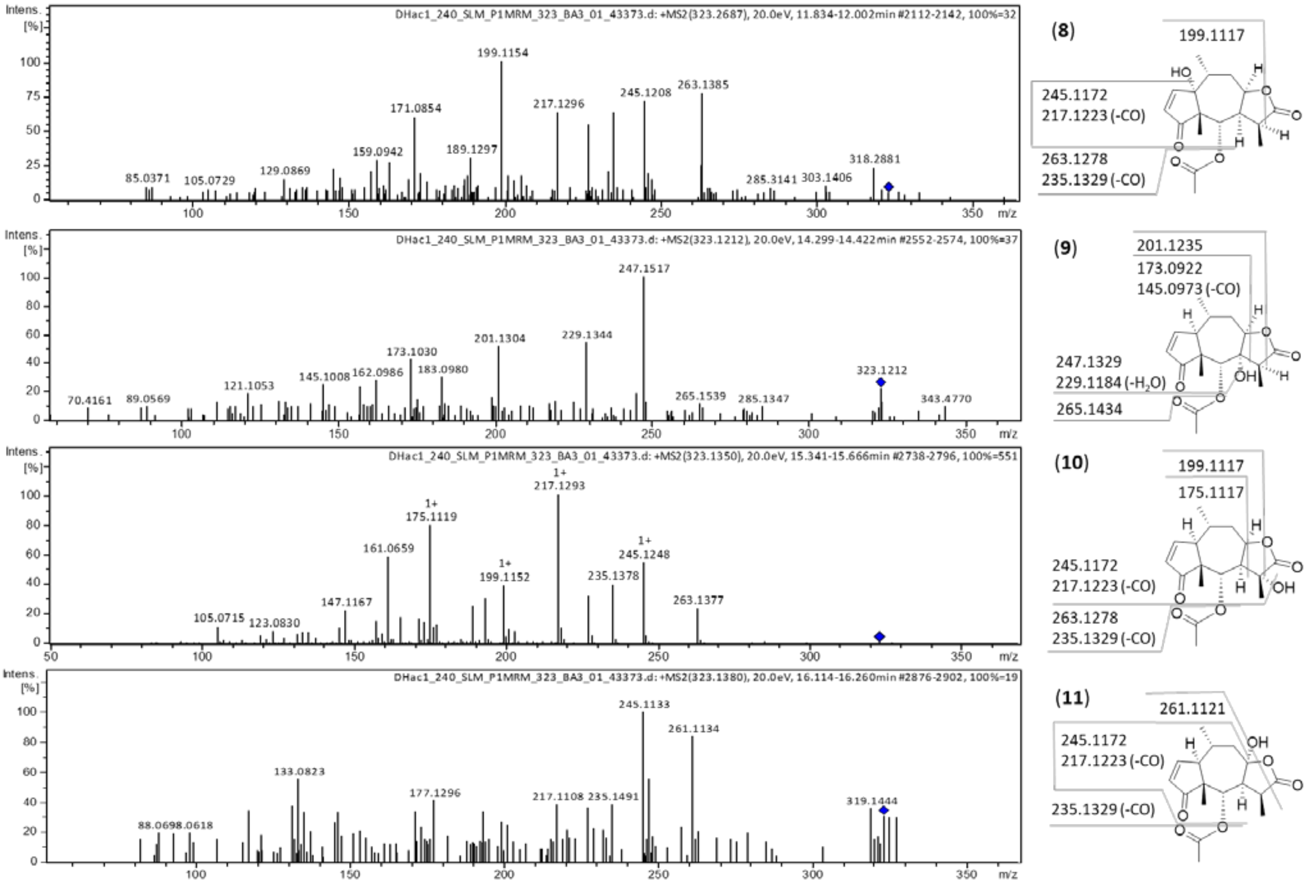

(**12**) DHac-$(OH)_2$

Metabolite (**12**) with $[M+H]^+$ at *m/z* 339.1438, $[M+Na]^+$ *m/z* 361.1258 and $[M+K]^+$ at *m/z* 377.0997 is identified as di-hydroxylated DHac. The fragment at *m/z* 279.1735 results from loss of acetic acid and the ones at *m/z* 261.1257 and *m/z* 243.1033 from one and two additional dehydrations, respectively. This metabolite is clearly a di-hydroxylated derivative of DHac, and most likely represents a combination of the most abundant mono-hydroxylated metabolites (**8** and **10**): DHac-1,11$(OH)_2$. If (**12**) would have been hydroxylated at C-7, loss of 58.0055 Da as observed for (**9**) would have been expected and with a possible hydroxylation at C-8, cleavage of 62.0368 Da as observed for (**11**) would have been expected. In contrary, loss of 60.0265 Da was detected as observed for (**8**) and (**10**). Fragment *m/z* 251.1357 is formed by loss of acetic acid and CO, *m/z* 233.1192 results from dehydration of the latter and *m/z* 205.1264 from additional loss of CO. The fragment *m/z* 173.0707 results if the hydroxide at C-11 is cleaved as hydroxyacetylene (C_2H_2O, as discussed for (**10**)) in case of (**12**) after dehydration at C-1/C-10.

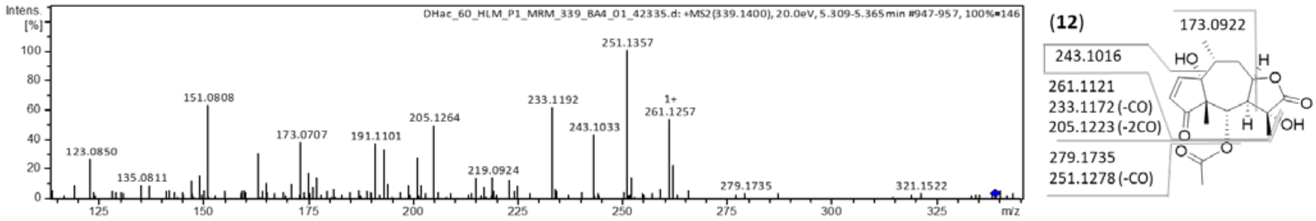

(**13**-**14**) DHac-H_2O-OH

Two metabolites with $[M+H]^+$ at *m/z* 341.1595, $[M+Na]^+$ at *m/z* 363.1414 and $[M+K]^+$ at *m/z* 379.1154 were detected with retention times 5.2 min and 5.7 min, respectively. These metabolites can tentatively be assigned to result from a combination of the previously described water conjugates and hydroxides. For metabolite (**13**), the fragment at *m/z* 295.0644 results from loss of formic acid as discussed for (**6**) and leads to the conclusion that (**13**) is hydrolysed at the lactone moiety. Another interesting fragment is *m/z* 265.1464 that might result from neutral loss of 58.0055 Da followed by dehydration as discussed for (**9**). This implies that (**13**) is hydroxylated at C-7 (DHac-7OH-12H_2O). Additional dehydration results in *m/z* 247.1343 followed by typical DHac fragments (*m/z* 229.1123, *m/z* 219.1410 and *m/z* 173.0927).

For metabolite (**14**) the intense fragment at *m/z* 175.1088 implies the involvement of the most abundant hydroxide (**10**) (as discussed for (**10**)). Since no fragment at *m/z* 295.1540 is formed for (**14**), water is most likely conjugated at C-2 like for the most abundant water conjugate (**5**; see above). For this combination (DHac-2H_2O-11OH), dehydration is expected twice, which was confirmed by MS/MS analysis of (**14**): *m/z* 281.1327 results from loss of acetic acid, while *m/z* 263.1238 and *m/z* 245.1130 result from the former by one and two further dehydrations, respectively. Subsequent fragments at *m/z* 235.1294, *m/z* 227.1038 and *m/z* 217.1192, are typical Hac fragments (as discussed for metabolites (**8**-**11**)).

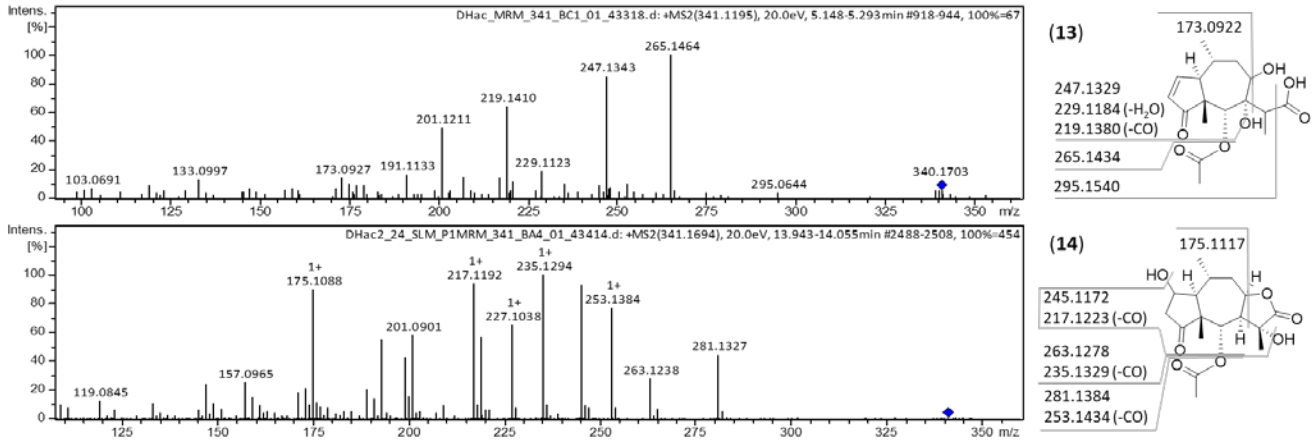

(**15**) Hac

Metabolite (**15**) ($[M+H]^+$ at 305.1384, $[M+Na]^+$ at 327.1203 and $[M+K]^+$ at 343.0942) was formed in minor amounts. For that reason, the MS/MS spectra is not very expressive. Nevertheless, the fragments *m/z* 245.1324 and *m/z* 199.1175 are typical Hac fragments as discussed in the main part of the article. Furthermore, retention time (Rt = 7.1 min) and accurate mass of the pseudo-molecular ions of (**15**) are in agreement with the isolated Hac. It has been excluded that metabolite (**15**) was a contamination by carry-over from the experiments with Hac.

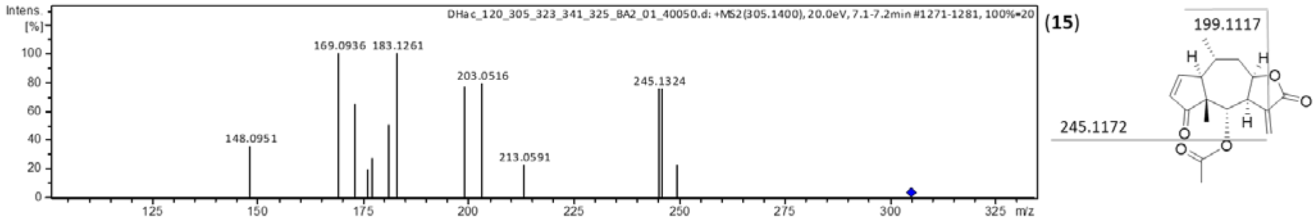

(**16**, **17**) Hac-$(GSH)_2$

For Hac, two metabolites with $[M+H]^+$ at *m/z* 919.3060 were detected. The mass difference to Hac (614.1676 Da) can be explained by addition of GSH at both Michael acceptors. Similar to the GSH mono-conjugates of Hac and DHac, a small second peak with the same accurate mass and fragmentation pattern elutes after the main peak indicating the presence of two stereoisomers. Stereochemistry of the spontaneously formed major Hac-GSH-di-conjugate was previously determined by NMR analysis as Hac-2β,13(11α*H*)$(GSH)_2$ [16]. Since metabolite (**16**) predominates, it is most likely the Hac-2β,13(11α*H*)$(GSH)_2$ stereoisomer. Consequently, (**17**) is the Hac-2α,13(11α*H*)$(GSH)_2$ stereoisomer. This consideration is consistent with the classification of DHac and Hac GSH mono-conjugates, for which the 2β-GSH-conjugate (major peak) elutes first, followed by a minor peak of the 2α-GSH-conjugate. The fragment at *m/z* 790.2652 (**16**) results from neutral loss of glutamic acid.

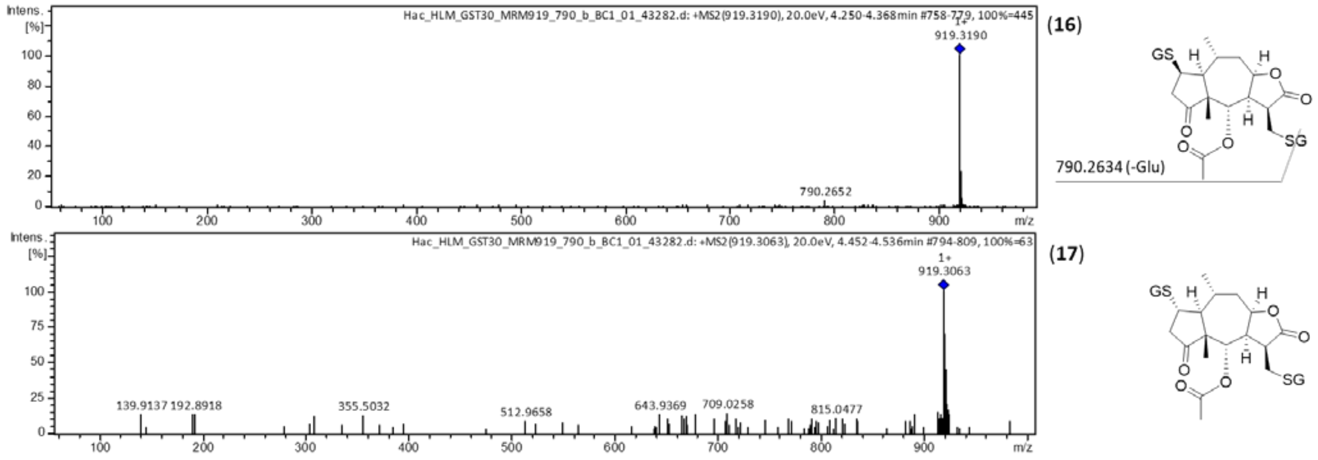

(**18**) Hac-GSH-CysGly

Metabolite (**18**) with $[M+H]^+$ at *m/z* 790.2634 was identified as Hac-GSH-CysGly, probably resulting from cleavage of glutamic acid from a GSH-di-conjugate. GSH-conjugation at C-2 was described to be reversible whereas GSH-conjugation at C-13 is more stable [34]. For this reason, the (reversible) conjugation of GSH is most likely at C-2 and the degradation to the dipeptide (Cys+Gly) occurred most likely at C-13 (Hac-2βGSH-13(11α*H*)CysGly). The fragment at *m/z* 601.0195 is formed by loss of acetic acid and glutamic acid, leading to a cysteine-glycine di-conjugate. The fragment *m/z* 439.0863 might result by neutral loss of glycine and ammonia from the latter (at C-2 and C-13) and additional demethylation. The fragment *m/z* 348.0749 results from cleavage of acetic acid, GSH, glycine and water starting from the parent ion at *m/z* 790.0720.

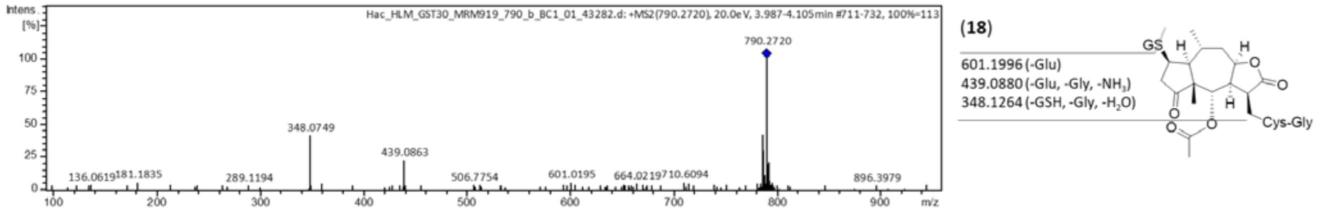

(**19**-**22**) Hac-GSH

Four metabolites with $[M+H]^+$ at *m/z* 612.2222 were identified as GSH mono-conjugates of Hac. The spontaneous formation of Hac-2βGSH and Hac-13(11α*H*)GSH without Hac-2αGSH and Hac-13(11β*H*)GSH were reported previously [16]. Furthermore, GSH-conjugation was favoured at the cyclopentenone moiety (compared to the exocyclic methylene group). For that reason, the major peak (**19**) was identified as Hac-2βGSH, followed by the stereoisomer Hac-2αGSH (**20**). The second largest of the mentioned metabolite peaks was identified as Hac-13(11α*H*)GSH (**21**) followed by the corresponding stereoisomer Hac-13(11β*H*)GSH (**22**). Comparability of the MS/MS spectra of (**19**) with (**20**) and (**21**) with (**22**) support the assumption that (**19**) and (**20**) as well as (**21**) and (**22**) are stereoisomers, respectively. In agreement with the other 2β- and 2α-conjugates (see above), Hac-2βGSH elutes earlier than Hac-2αGSH. In case of (**21**) and (**22**) the assignment is based on the fact that (**21**) (11a*H*) is formed as major adduct with unambiguous assignment of stereochemistry as reported earlier [34], whereas the minor epimer (**22**) found here for the first time was not detected at this time. Fragments of GSH (*m/z* 308.0891 (**19**), *m/z* 308.0898 (**20**), *m/z* 179.0491 (**19**), *m/z* 179.0462 (**20**), and *m/z* 179.0326 (**22**)) as well as fragments resulting from neutral loss of glutamic acid (*m/z* 483.1844 (**21**) and *m/z* 483.1795 (**22**)) or glycine (*m/z* 537.1969 (**21**)) and the core fragment (*m/z* 245.1185 (**19**)) are formed.

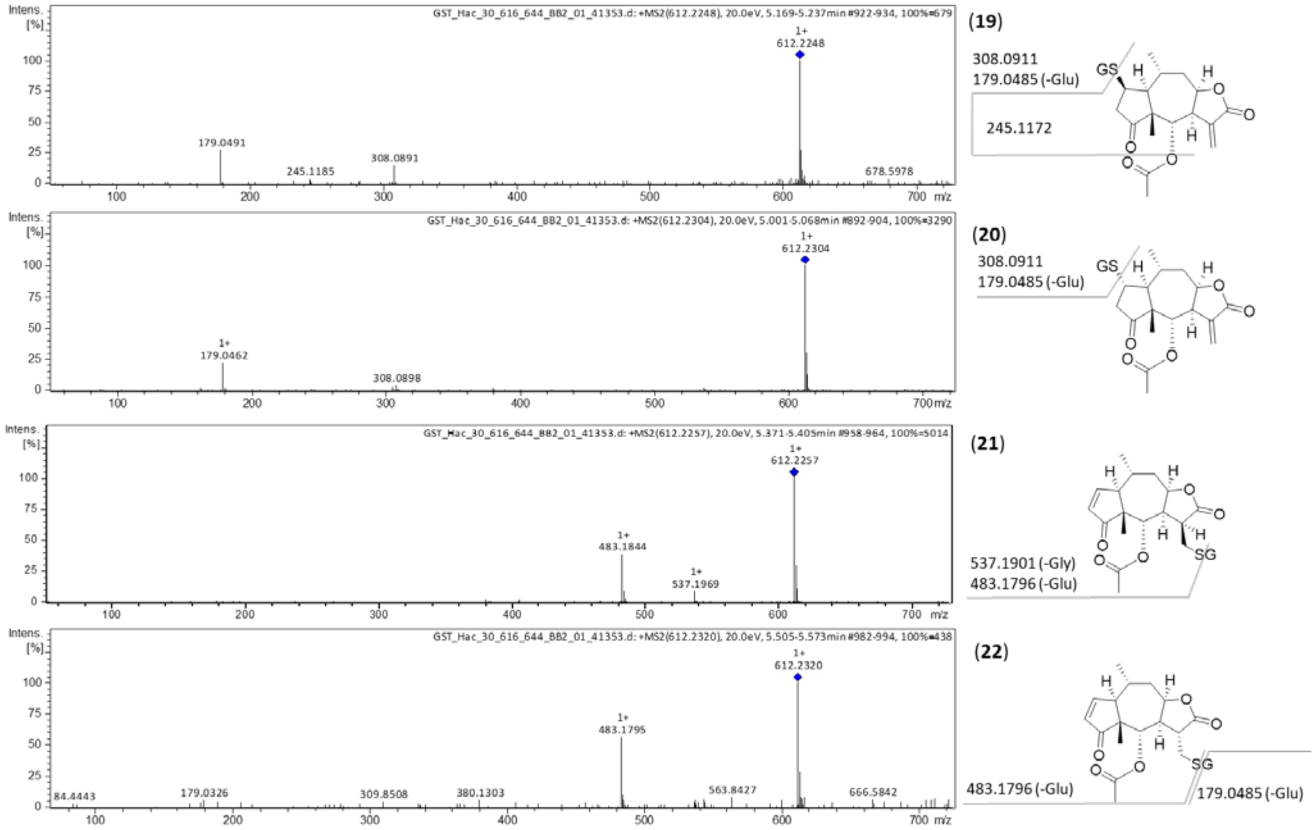

($\mathbf{23}$) Hac-GSH-H_2O

Metabolite (**23**) with $[M+H]^+$ at *m/z* 630.2327 was identified as Hac conjugated with GSH and water. As GSH-conjugation is reversible at C-2 and more stable at C-13 [34], it is most likely that GSH is conjugated a C-13 and water at C-2, resulting in the depicted tentative structure (Hac-2H_2O-13(11α*H*)GSH). Neutral loss of glutamic acid resulted in *m/z* 501.1892, loss of glycine in *m/z* 555.1891. Protonated GSH forms the fragment at *m/z* 308.0895.

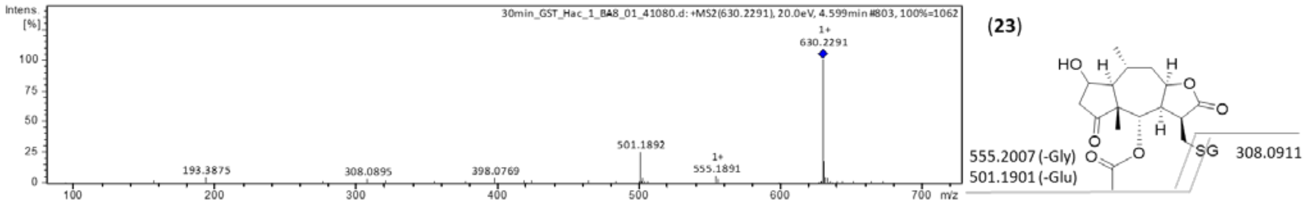

(**24**, **25**) Hac-H_2O-CH_3-GSH

Two metabolites were identified as Hac conjugated with GSH and water and additional methylation ($[M+H]^+$ at *m/z* 644.2484). A corresponding metabolite of Hac after addition of water and further methylation (**35**) was also detected (see below). *O*-methylation is a common metabolism reaction and the hydroxy group resulting from addition of water is most probably the methylation target in this case. Moreover, neutral loss of glutamic acid (leading to *m/z* 515.2061 (**24**) and *m/z* 515.2457 (**25**)) as well as neutral loss of glycine (resulting in *m/z* 569.2361 (**24**) and *m/z* 569.2266 (**25**)) show that the tripeptide is not modified. Both metabolites have a small retention time difference (± 0.1 min) indicating that (**24**) and (**25**) are stereoisomers. In comparison, the GS-mono-conjugates (**19**) and (**21**) with GSH conjugated at C-2 or C-13 differ in Rt ± 0.4 min supporting the assumtion that GSH in (**24**) and (**25**) is conjugated at the same position (C-13). Both are minor metabolites but in comparison

with (**24**), (**25**) is formed in higher amounts leading to the (tentative) conclusion that (**25**) is the sterically favoured Hac-2βH$_2$O-CH$_3$-13(11α*H*)GSH and (**24**) the Hac-2αH$_2$O-CH$_3$-13(11α*H*)GSH.

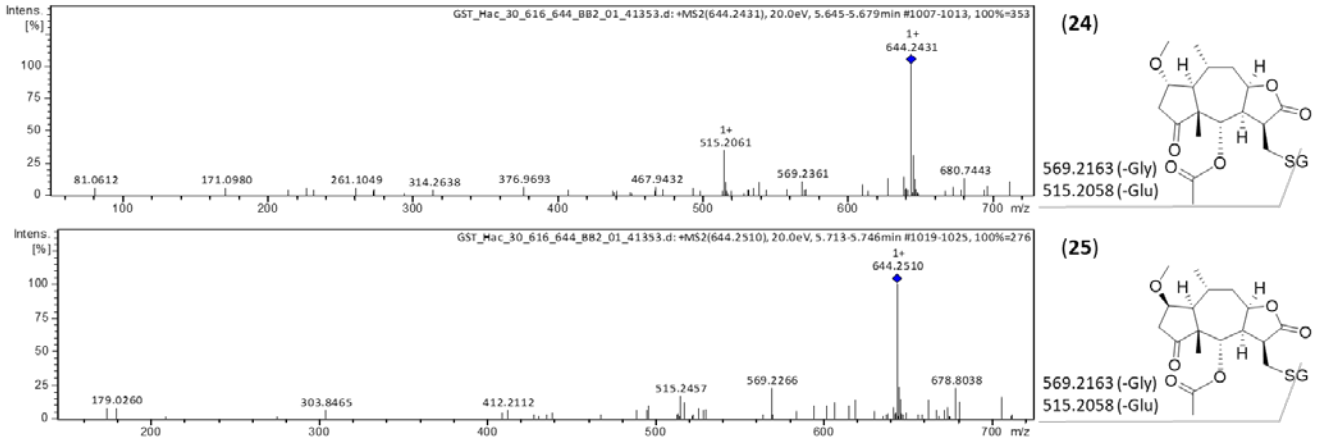

(**26**, **27**) Hac-CysGly

Two metabolites were identified as Hac-CysGly conjugates with [M+H]$^+$ at *m/z* 483.1796 resulting most likely from GSH conjugation (**19**-**22**) and cleavage of Glu. The fragments at *m/z* 466.1612 (**26**) and *m/z* 466.1691 (**27**) result from neutral loss of ammonia (NH$_3$, 17.0265 Da). Additional loss of acetic acid results in *m/z* 406.1438 (**26**) and *m/z* 406.1429 (**27**). This fragment is also formed in case of Hac-13GSH conjugates (**21**) and (**22**) but not in the Hac-2GSH conjugates (**19**) and (**20**). For that reason (**26**) and (**27**) are considered as Hac-CysGly conjugates at C-13. Since (**26**) predominates, it is concluded that it is the 11α epimer (Hac-13(11α*H*)CysGly) and (**27**) the 11β epimer (Hac-13(11β*H*)CysGly). Both metabolites share the core fragment *m/z* 245.1227 (**26**) and *m/z* 245.1337 (**27**) with the parent compound and a fragment formed by neutral loss of acetic acid at *m/z* 423.1571 (**26**).

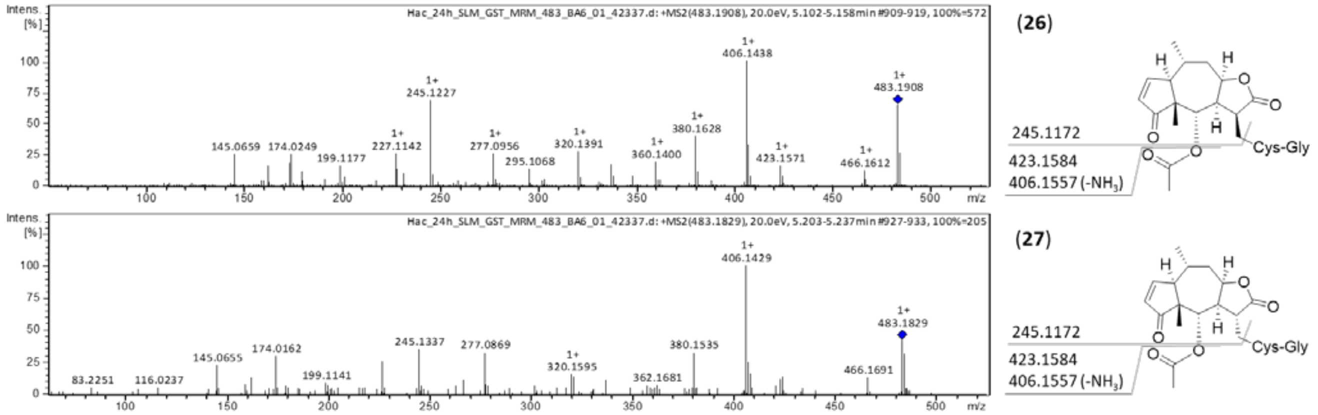

(**28**-**31**) Hac-CysGly-H$_2$O

Four metabolites with [M+H]$^+$ at *m/z* 501.1901 were identified as Hac conjugated with CysGly like (**26**) and (**27**) and an additional molecule of water added. Cleavage of acetic acid results in the fragments *m/z* 441.1787 (**28**), *m/z* 441.1720 (**29**), *m/z* 441.1709 (**30**) and *m/z* 441.1768 (**31**), respectively. Additional neutral loss of the dipeptide gives *m/z* 263.1286 (**28**), *m/z* 263.1255 (**29**), whereas *m/z* 179.0460 (**29**) and *m/z* 179.0488 (**30**) represent the protonated dipeptide CysGly. The fragments *m/z* 406.1227 (**28**) and *m/z* 406.1305 (**30**) result from neutral loss of water, acetic acid and ammonia (NH$_3$). Since GSH-conjugates are more stable at C-13 and Hac-CysGly metabolites at C-13 (**26**, **27**) were found, it is most likely that (**28**-**31**) result from water conjugation at

(**26**) and (**27**). This consideration is supported by the fragments at *m/z* 406.1227 (**28**) and *m/z* 406.1305 (**30**) which are only formed in case of Hac-GSH conjugates at C-13. Of the minor metabolites (**28**-**31**), (**29**) is the most abundant, leading to the conclusion that (**29**) is the Hac-(11α*H)*13CysGly conjugate with a water molecule added to C-2 (2βOH) (Hac-2βOH-(11α*H)*13CysGly). The second strongest metabolite is (**30**); probably resulting from (**27**) and thus the Hac-(11β*H*)13CysGly conjugate with a 2βOH due to water addition (Hac-2βOH-(11β*H*)13CysGly). The small metabolites (**28**) and (**31**) are most likely the corresponding 2αOH water adducts (Hac-2αOH-(11α*H)*13CysGly and Hac-2αOH-(11β*H*)13CysGly). This consideration is supported by the sequence of retention times where (**26**) as well as (**28**) and (**29**) with 11α*H*-configuration elute earlier than (**27**), (**30**) and (**31**) with 11β*H*-configuration.

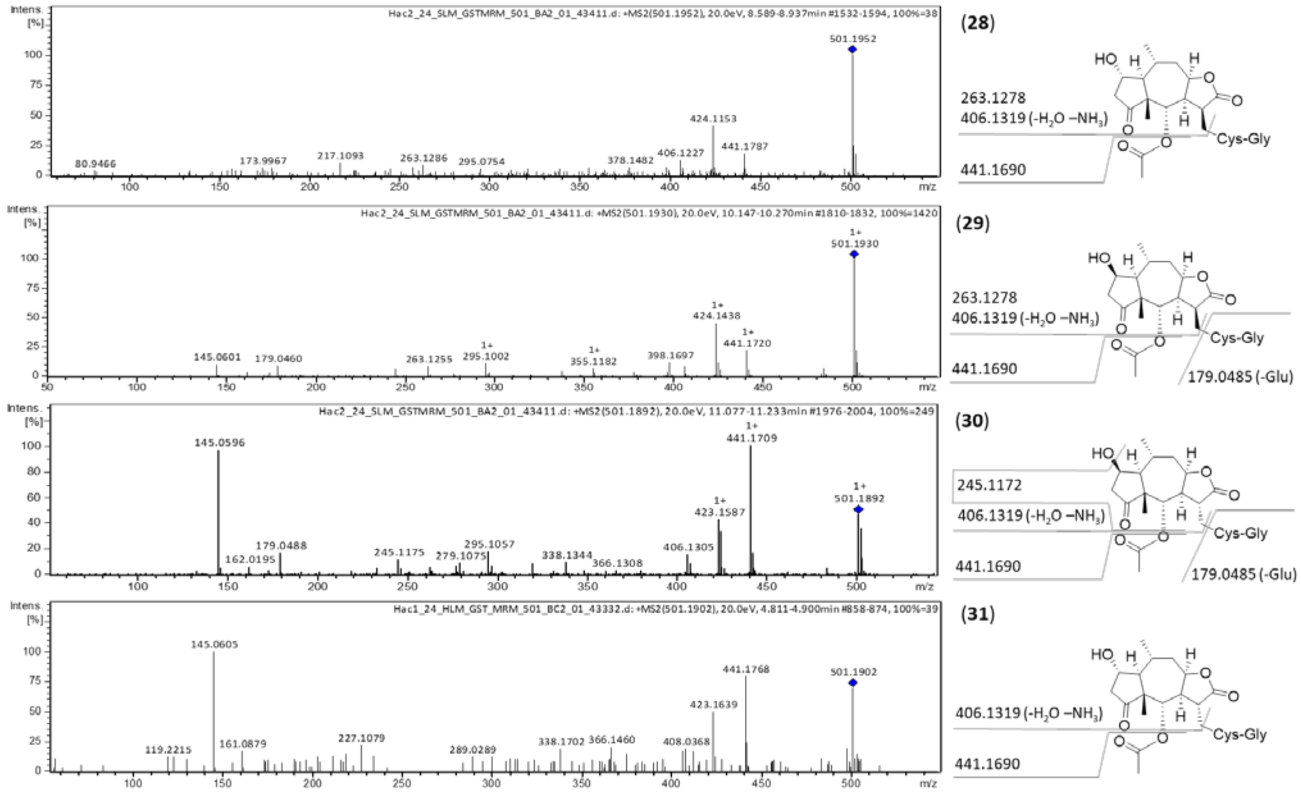

(**32**) Hac-Cys

One Hac-Cys conjugate was identified with $[M+H]^+$ at *m/z* 426.1581. After neutral loss of ammonia, *m/z* 409.1477 results. The fragment *m/z* 366.1543 is formed by loss of acetic acid and *m/z* 245.1259 as well as *m/z* 173.0760 represent typical fragments of Hac. As GSH conjugates are more stable at the exocyclic methylene group, it can be concluded that the degradation products resulting from loss of amino acids are derived from the conjugates at this position. Besides the degradation of GSH conjugates to Cys conjugates, the formation of Hac-13(11α*H*)Cys conjugates is also known with free Cys. In this process, Cys is much more likely to react with the exocyclic methylene group than with the cyclopentenone moiety of helenalin [34]. This conjugation was also observed in the phase I experiment without GSH and GST supplement. Since only one Cys conjugate was found, it can be assumed that Cys was added at the exocyclic methylene group. In agreement with the previous literature [34], this would also explain why no Cys conjugate was formed with DHac, which has no exocyclic methylene group.

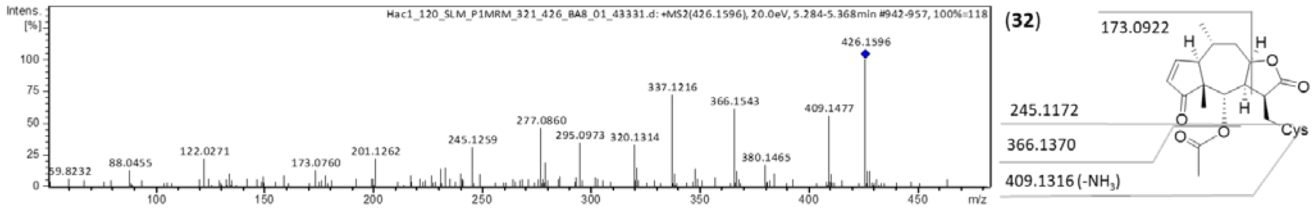

(**33**-**34**) Hac-H_2O

Two metabolites were identified as Hac water adducts with $[M+H]^+$ at *m/z* 323.1490, $[M+Na]^+$ at *m/z* 345.1309 and $[M+K]^+$ at *m/z* 361.1048. It is considered most likely, that water is added in Michael addition at C-2 or at C-13, respectively. With MS/MS fragmentation the water adducts can not be differentiated because water is removed in the first steps. Both water adducts show fragments resulting from loss of acetic acid (*m/z* 263.1272 (**33**), *m/z* 263.1260 (**34**)), additional loss of water (*m/z* 245.1183 (**33**), *m/z* 245.1182 (**33**)), further loss of CO (*m/z* 217.1217 (**33**), *m/z* 217.1214 (**34**)) and dehydration of the core fragment *m/z* 245.1183 or *m/z* 245.1182 resulting in *m/z* 199.1113 (**33**) and *m/z* 199.1109 (**34**). Considering retention times (Rt = 6.0 min (**33**) and Rt = 6.3 min (**34**)) and the fact that (**34**) is very similar with (**13**) which elutes at 6.3 min, it can be concluded that (**34**) is the water adduct at C-13 (Hac-13H_2O) and (**33**) at C-2 (Hac-2H_2O).

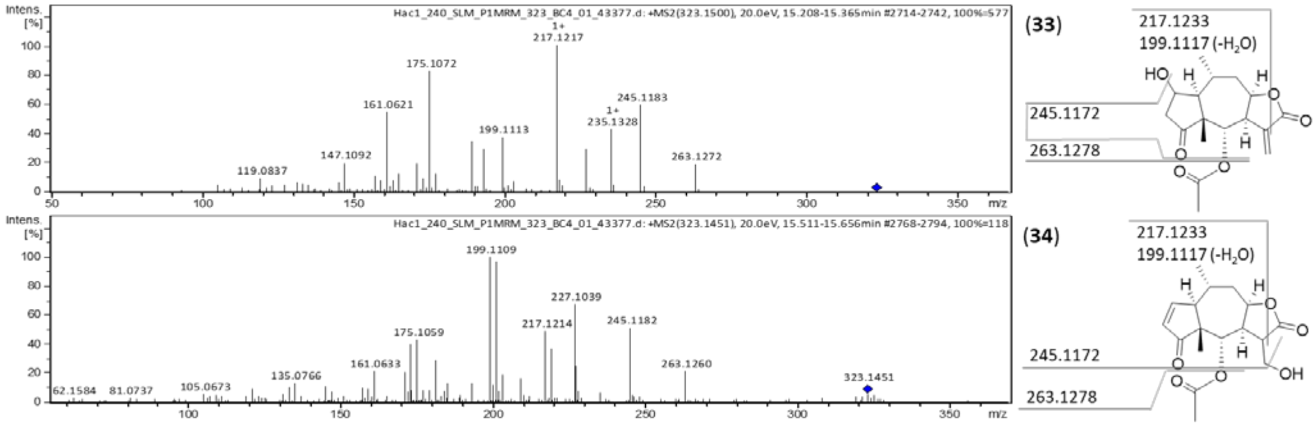

(**35**) Hac-H_2O-CH_3

One methylated water adduct with $[M+H]^+$ at *m/z* 337.1646, $[M+Na]^+$ at *m/z* 359.1465 and $[M+K]^+$ at *m/z* 375.1204 was detected. With the two different water adducts described before, two methylated water adducts are possible. In analogy to DHac, where also one methylated water adduct was found and no water adduct at C-13 is possible, it is most likely that (**35**) is the methylated water adduct at C-2 (Hac-2H_2O-CH_3). Similar Hac-2H_2O-CH_3 conjugates were also detected in combination with GSH conjugation (cf. (**24**-**25**)). Regarding fragmentation, *m/z* 277.1582 (loss of acetic acid), *m/z* 259.1284 (additional dehydration) and *m/z* 245.1200 (loss of acetic acid and methanol) are observed.

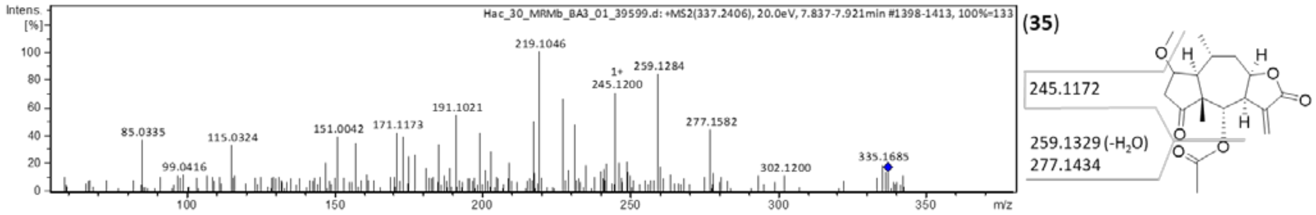

(**36**-**37**) Hac-OH

Two metabolites with $[M+H]^+$ at *m/z* 321.1333, $[M+Na]^+$ at *m/z* 343.1152 and $[M+K]^+$ at *m/z* 359.0891 were detected. The mass difference of 15.9949 Da compared with Hac indicates that these metabolites are hydroxides or epoxides. For an epoxide the loss of 15.9949 Da in the fragmentation pattern from *m/z* 261.1121 to *m/z* 245.1172 is expected. On the contrary, for hydroxides loss of acetic acid and water resulting in fragments *m/z* 261.1121 and *m/z* 243.1016, is anticipated.

As discussed for DHac hydroxides (**8**-**11**), hydroxylation is likely at tertiary carbons in α position to functional groups. In contrast to DHac, C-11 of Hac is not accessible for hydroxylation because it is a quaternary carbon. Instead, C-7 is a tertiary carbon in α-position to the α-methylene-γ-lactone moiety and therefore a likely hydroxylation site of Hac. For metabolite (**36**) fragments at *m/z* 263.1509 and *m/z* 245.1263 were detected. The fragment at *m/z* 263.1509 results from loss of 57.9805 Da, the fragment *m/z* 245.1263 by subsequent dehydration. Usually, neutral loss of acetic acid ($C_2H_4O_2$, 60.0265 Da) is the first step in Hac and DHac fragmentation. This process should be different if C-7 is hydroxylated, since no proton is available at C-7 to complete the acetic acid molecule. Thus, a loss of $C_2H_2O_2$ (58.0055 Da) could result in such cases (and is also observed in case of the hydroxylated metabolite of DHac (**9**), see above). Hence, (**36**) is assumed to be a hydroxide at C-7. For this structure (Hac-7OH), the fragments *m/z* 227.1017 ($[M+H-C_2H_4O_2-H_2O]^+$), *m/z* 217.1370 ($[M+H-C_2H_4O_2-CO]^+$) and 199.1140 ($[M+H-C_2H_4O_2-H_2O-CO]^+$) are in common with Hac.

For metabolite (**37**) the fragments *m/z* 243.1151, *m/z* 215.1084 and *m/z* 197.1011 are formed, indicating loss of water and thus leading to the conclusion that this metabolite is also a hydroxide. Comparable to DHac, three other tertiary carbons in in α position to functional groups (C-1, C-6 and C-8) might be targets for hydroxylation. C-6 can be excluded for (**37**) because fragments at *m/z* 261.1121 followed by *m/z* 245.1172 (by deoxygenation) are expected. For a hydroxide at C-8 neutral loss of 62.0368 Da would be expected as observed for the corresponding DHac hydroxide (**11**). For a hydroxide at C-1, fragments at *m/z* 261.1101 ($[M+H-C_2H_4O_2]^+$), *m/z* 243.1151 ($[M+H-C_2H_4O_2-H_2O]^+$), *m/z* 233.1098 ($[M+H-C_2H_4O_2-CO]^+$), *m/z* 215.1084 ($[M+H-C_2H_4O_2-H_2O-CO]^+$), and *m/z* 197.1011 ($[M+H-C_2H_4O_2-2H_2O-CO]^+$) are reasonable so that metabolite (**37**) is assigned this tentative structure (Hac-1OH).

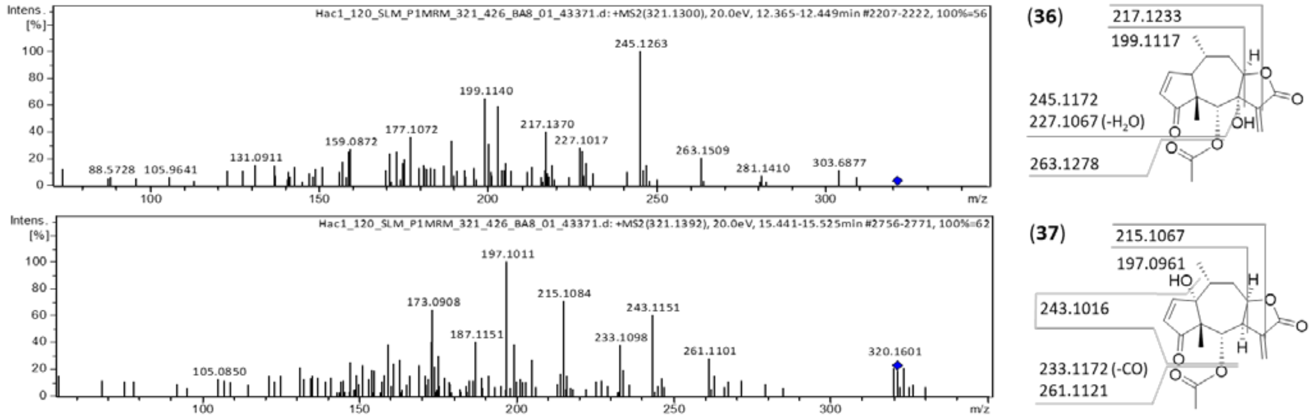

(**38**) Hac-H_2O-OH

Similar to DHac, one hydroxylated water conjugate was detected with $[M+H]^+$ at *m/z* 339.1438, $[M+Na]^+$ at *m/z* 361.1258 and $[M+K]^+$ at *m/z* 377.0997. Loss of acetic acid resulted in *m/z* 279.1251, additional dehydration in *m/z* 261.1165 a second dehydration in *m/z* 243.1080 leading to the conclusion that (**38**) contains two hydroxy groups and is formed by hydration of one of the hydroxides (or by hydroxylation of one of the water adducts). Two hydroxides (**36**) and (**37**) and two water adducts (**33**) and (**34**) were detected for Hac. The combination of fragments *m/z* 279.1251 ($[M+H-60.0265\ Da]^+$) and *m/z* 261.1165 (additionally dehydrated) confirms the

position of the hydroxide at C-1 because in case of a Hac hydroxide at C-7 like (**36**), loss of 58.0055 Da instead of 60.0265 would be observed. To distinguish between the water adducts, fragmentation is not helpful because both such adducts (**33** and **34**) share the same fragments. In comparison with DHac-7OH-12H_2O (**13**, Rt = 5.2 min) and DHac-2H_2O-11OH (**14**, Rt = 5.7 min), the retention time of Hac-H_2O-OH (**38**, Rt = 5.8 min) is much closer to the latter, in which the OH groups are located at the far ends of the molecule. Therefore, with due caution, we hypothesize that (**38**) is most likely a combination of (**37**) and (**34**), i.e. Hac-1OH-13H_2O with both functional groups at opposite sites of the molecule.

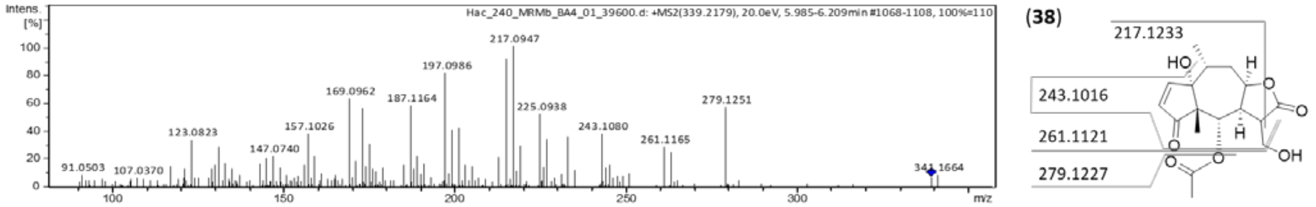

(**39**) DHac

In Hac samples one metabolite with $[M+H]^+$ at *m/z* 307.1540, $[M+Na]^+$ at *m/z* 329.1359 and $[M+K]^+$ at *m/z* 345.1099 was detected with typical DHac MS/MS fragments. Loss of acetic acid leads to the fragment at *m/z* 247.1243 ($[M+H-C_2H_4O_2]^+$). Additional dehydration results in fragment *m/z* 229.1064, further fragmentation leads to the fragments *m/z* 201.1253 and *m/z* 173.1067 as described in the main text. The latter results in *m/z* 145.0964 by CO cleavage. It has been excluded that metabolite (**39**) was a contamination by carry-over from the experiments with DHac.

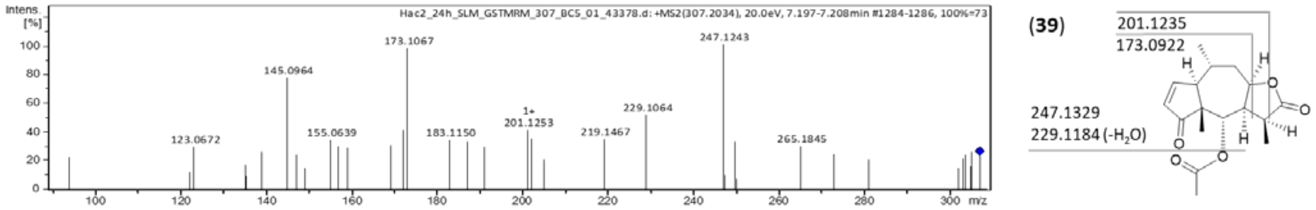

Supplement: Supplementary file 1 [file metabolites-12-00088-s001.zip › Supplementary_Materials_revised2.pdf]
